# Granulomatous Cheilitis: Successful Treatment of Two Recalcitrant Cases with Combination Drug Therapy

**DOI:** 10.1155/2014/509262

**Published:** 2014-10-15

**Authors:** Ambika Gupta, Harneet Singh

**Affiliations:** Department of Oral Medicine and Radiology, Pandit B.D. Sharma UHS (PGIDS), Rohtak, Haryana, India

## Abstract

Granulomatous cheilitis is a rare, idiopathic, inflammatory disorder which usually affects young adults. It is characterized by persistent, diffuse, nontender, soft-to-firm swelling of one or both lips. Various treatment modalities have been suggested. In spite of the best treatment, recurrence of the disease is very common. We report two cases of granulomatous cheilitis treated with a combination of steroids, metronidazole, and minocycline with no signs of relapse at one-year follow-up.

## 1. Introduction

Orofacial granulomatosis comprises a group of diseases characterized by noncaseating granulomatous inflammation affecting the soft tissues of the oral and maxillofacial region [1]. The term, introduced by Wiesenfeld et al. in 1985, includes Melkersson-Rosenthal syndrome and cheilitis granulomatosa of Miescher [2]. Melkersson-Rosenthal syndrome manifest itself as a triad of recurrent or persistent lip or facial swelling, recurrent, partial, or complete facial paralysis, and fissured tongue [3, 4]. Cheilitis granulomatosa of Miescher is characterized by swelling restricted to the lips [5]. Granulomatous cheilitis is considered a monosymptomatic form of Melkersson-Rosenthal syndrome by some clinicians. The etiology of this disease is unclear, but the condition has been linked to an abnormal immune reaction. The available therapeutic options provide only limited and temporary remissions. Two cases of granulomatous cheilitis are being reported, who showed an excellent and sustained response to combination of intralesional steroids, metronidazole, and minocycline.

## 2. Case 1

**Figure 1:**
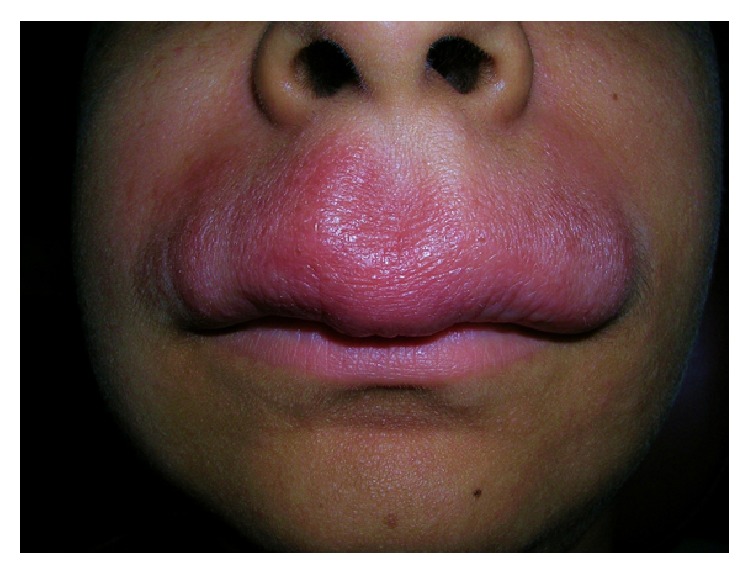
Pretreatment view of the swelling in the first patient showing diffuse, erythematous swelling of upper lip.

**Figure 2:**
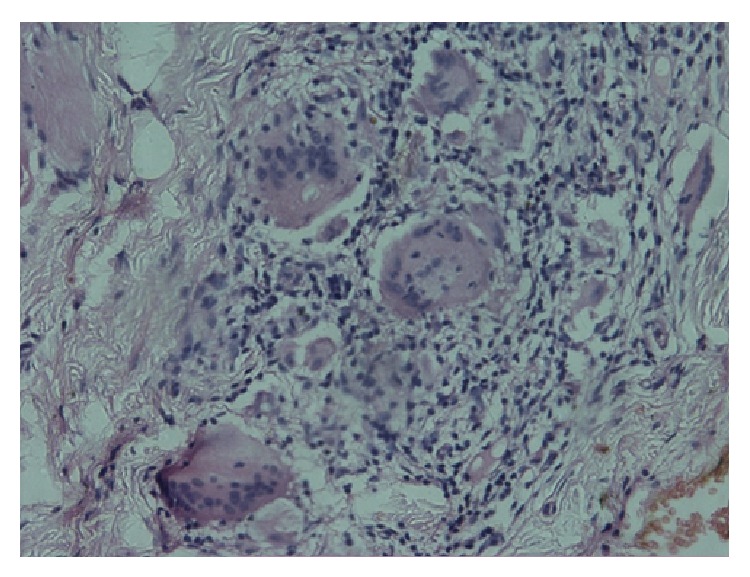
Histopathological pictures showing Langhans giant cells, epithelioid cells, lymphocytes, and neutrophils.

**Figure 3:**
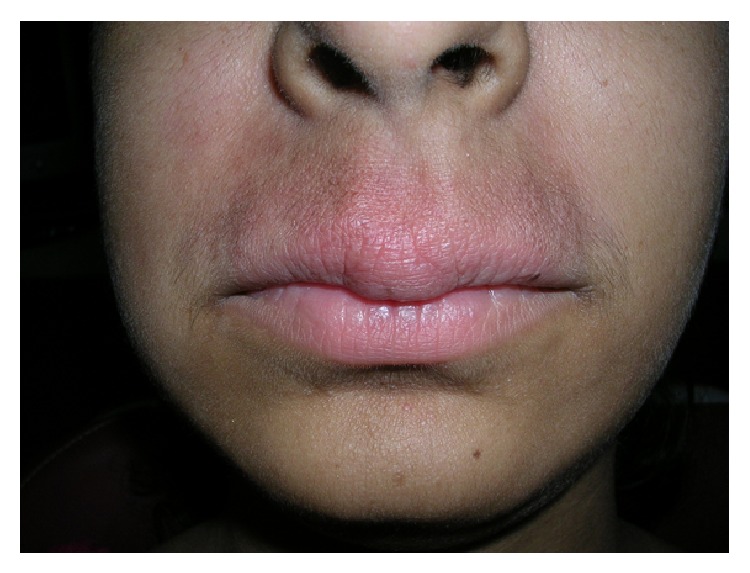
Posttreatment view of the first patient with marked improvement of swelling and erythema.

A 17-year-old female reported in an outdoor department of Oral Medicine at Government Dental College, Rohtak, with a 2-year history of persistent asymptomatic swelling of the upper lip and occasional gingival swelling (Figure 1). Her medical history was noncontributory. There was no history suggestive of abdominal cramps, diarrhea, fatigue, weight loss, or any other gastrointestinal disorders. Systemic examination did not reveal any abnormalities. Examination revealed a nontender, diffuse, firm swelling of the upper lip. The surrounding facial skin showed diffuse erythematous swelling. The surface of the lip was smooth with no signs of scabs, bleeding, or exudation. No fissuring of the tongue, oral ulcers, or hypertrophy of the oral mucosa was noticed. There was no palsy of facial muscles. The patient had received intralesional triamcinolone injections in the past with temporary remissions and recurrences of the swelling. A chest radiograph, complete haemogram, erythrocyte sedimentation rate, serum folate, iron, and vitamin B12 levels, serum levels of angiotensin-converting enzyme, were ordered, which were in normal range. The tuberculin skin test for tuberculosis was negative. Ultrasonography of the upper lip revealed a mildly increased vascularity in the region. The diagnosis of cheilitis granulomatosa was confirmed on a histopathological examination, which revealed Langhans type giant cells, epithelioid cells, lymphocytes, and few neutrophils (Figure 2). We decided to treat her with a combination of intralesional weekly injections of triamcinolone acetonide 10 mg/mL in the upper lip for 4 weeks, along with oral metronidazole 400 mg three times a day and oral minocycline 100 mg daily. There was a significant improvement in the labial swelling and erythema after 15 days of treatment. The gingival swelling also subsided after 20 days. After one month, metronidazole was withdrawn and minocycline was continued on alternate days for an additional one month. A recurrence of swelling of the upper lip was noticed after 4 months, which subsided with an injection of intralesional triamcinolone acetonide solution 10 mg/mL. In a 1-year follow-up, there was no further recurrence (Figure 3).

## 3. Case 2

**Figure 4:**
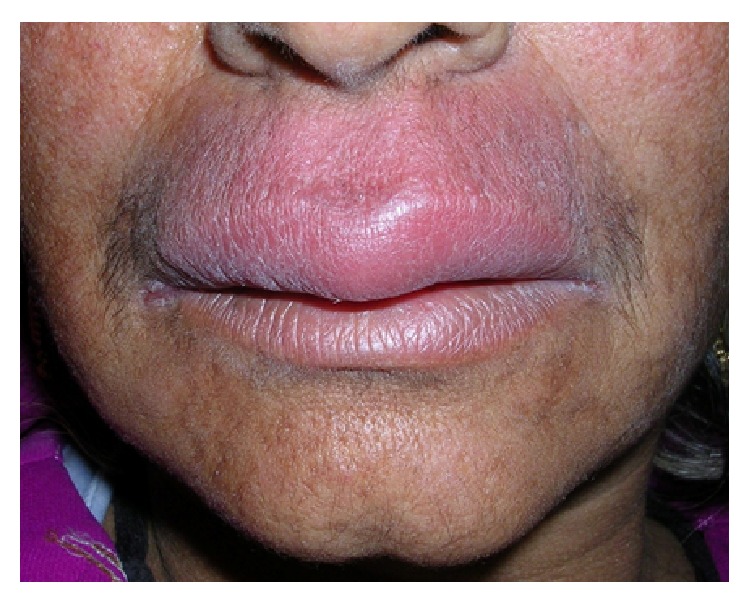
Pretreatment photograph of second patient showing diffuse swelling of upper lip.

**Figure 5:**
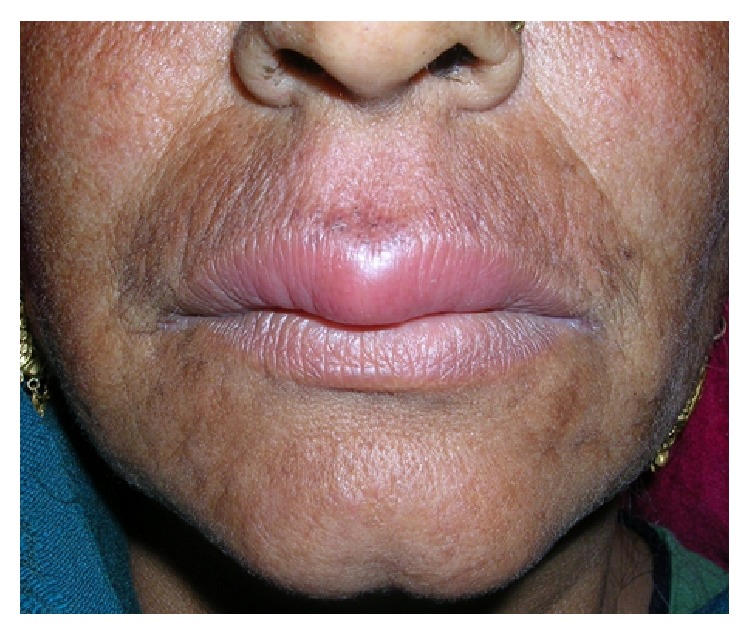
Posttreatment photograph of the second patient after 1 year.

A 58-year-old female reported with a 6-month history of asymptomatic swelling of the upper lip. Her medical history was noncontributory. She reported no intestinal problems that would suggest Crohn's disease, nor did she complain of chronic fatigue. There was no history of tuberculosis. Examination revealed a nontender, diffuse, erythematous, and firm- to-soft swelling of the upper lip. The surface of the lip was dry and smooth (Figure 4). There were no appreciable changes in the tongue or any ulceration of the oral mucosa. All the investigations done to rule out other differential diagnoses were within normal ranges. These included chest radiography and assessment of serum levels of angiotensin-converting enzyme for sarcoidosis; complete blood count, erythrocyte sedimentation rate and serum levels of folic acid, iron and vitamin B12 for Crohn's disease; and tuberculin skin test and chest radiography for tuberculosis. Histopathological findings revealed perivascular lymphocytic infiltration and noncaseating granulomas that were not well formed. Ziehl-Neelsen and periodic acid-Schiff (PAS) staining yielded negative results. We started the treatment with intralesional triamcinolone injections in the upper lip without any improvement. So, we decided to treat her with the same combination of intralesional triamcinolone acetonide 10 mg/mL, oral metronidazole 400 mg three times a day, and oral minocycline 100 mg daily, as in the previous case. We noticed a significant improvement in the labial swelling after 1 month of treatment. After one month, metronidazole was withdrawn and minocycline was continued on alternate days for an additional one month. At 1-year follow-up, there was no sign of recurrence (Figure 5).

## 4. Discussion

The exact etiology of orofacial granulomatosis is unknown [6]. Several theories have been postulated, including infection, genetic predisposition, and allergy [7–9]. A monoclonal lymphocytic expression, secondary to the chronic antigenic stimulation, cytokine production leading to granulomas formation, and a cell-mediated hypersensitivity reaction have also been suggested [10].

The clinical features of orofacial granulomatosis are highly variable. The classical clinical presentation of cheilitis granulomatosa is recurrent labial swelling of one or both lips [11]. The swellings are soft-to-firm in its consistency and nontender and eventually become persistent. Sometimes, the swelling extends to the chin, cheeks, periorbital region, and eyelids [12]. Rarely, superficial amber colored vesicles, resembling lymphangiomas, may be seen [13]. Intraorally the disease may cause gingival hypertrophy, erythema, pain, and erosions. The predominant lesions are edema, ulcers, and papules. The tongue may develop fissures, edema, paresthesia, erosions, or taste alterations. Cobblestone appearance of buccal mucosa may be seen. The palate may have papules or hyperplastic tissue [13]. Both cases reported here had a persistent swelling of the upper lip with gingival enlargement in the first case.

Orofacial granulomatosis may occur as the oral manifestation of a systemic condition, such as Crohn's disease, Sarcoidosis, or more rarely Wegener's granulomatosis [14]. Other differential diagnoses include tuberculosis, leprosy, systemic fungal infections and foreign body reactions, amyloidosis, certain soft-tissue tumours, angioedema, minor salivary gland tumour, and Ascher's syndrome [11]. All these local and systemic conditions may be a diagnostic dilemma and must be excluded by appropriate clinical and laboratory investigations [6, 14]. In the present cases, as the history and initial investigations were not suggestive of any gastrointestinal involvement, an in-depth evaluation of the gastrointestinal system did not appear justified.

The management of granulomatous cheilitis becomes difficult in the absence of knowledge concerning its etiology. The treatment objectives are to improve the patient's clinical appearance and comfort. Although rare, spontaneous remission is possible [1]. The elimination of odontogenic infections may reduce the swelling in certain patients [15].

First-line treatment is local or systemic corticosteroids or both. Intralesional injections of triamcinolone 10–40 mg/mL are often helpful [16]. However, relapses are common, with the use of corticosteroids, and long-term treatment may be required. Other therapeutic measures have been reported in the literature, including hydroxychloroquine, methotrexate, clofazimine, metronidazole, minocycline, thalidomide, dapsone, and danazol [17–19]. Cheiloplasty is reserved for resistant cases or those complicated by a major lip deformation.

Coskun et al. have reported successful results with a combination of intralesional steroids and metronidazole [20]. Similarly, Stein and Mancini treated two children successfully, with a combination of oral prednisolone and minocycline [21]. Dar et al. used a combination of intralesional triamcinolone, metronidazole, and minocycline to treat a patient and observed a marked improvement in the lip swelling after one month treatment [22]. We also decided to follow the same regimen as tried by Dar et al. in our two cases.

We injected the patient weekly with intralesional triamcinolone acetonide solution 10 mg/mL in the upper lip (0.25–0.50 mL at three points) for 4 weeks and prescribed oral metronidazole tablets 400 mg, three times a day, and oral minocycline 100 mg daily for one month. A significant decrease in swelling was noticed in both the patients after a period of 15 days. After one month, intralesional steroid and metronidazole were discontinued. Minocycline, however, was continued in a dose of 100 mg on alternate days for the next month. The dose of minocycline was tapered to look out for any relapse and to get sustained results. Both patients were followed up regularly for a period of one year without any relapses. The complete remission of the swelling may be attributable to the potent anti-inflammatory action of the drug combination used here. The treatment was well tolerated by both patients with no evidence of any side effects.

## 5. Conclusion

Based upon our experience with the two reported cases, we agree with the observations of Dar et al. We also recommend that a combination of intralesional triamcinolone injection, along with oral metronidazole and minocycline, seems to be an effective remedy for successful and sustained response in granulomatous cheilitis. Further, randomized case control trials are needed for establishing a universally accepted protocol for management of cheilitis granulomatosa.
